# Host-Derived Cytotoxic Agents in Chronic Inflammation and Disease Progression

**DOI:** 10.3390/ijms24033016

**Published:** 2023-02-03

**Authors:** Jürgen Arnhold

**Affiliations:** Medical Faculty, Institute of Medical Physics and Biophysics, Leipzig University, Härtelstr. 16-18, 04107 Leipzig, Germany; juergen.arnhold@medizin.uni-leipzig.de

**Keywords:** cytotoxic agents, reactive species, transition metal ions, free heme, serine proteases, angiotensin II, matrix metalloproteases, chronic inflammation

## Abstract

At inflammatory sites, cytotoxic agents are released and generated from invading immune cells and damaged tissue cells. The further fate of the inflammation highly depends on the presence of antagonizing principles that are able to inactivate these host-derived cytotoxic agents. As long as the affected tissues are well equipped with ready-to-use protective mechanisms, no damage by cytotoxic agents occurs and resolution of inflammation is initiated. However, long-lasting and severe immune responses can be associated with the decline, exhaustion, or inactivation of selected antagonizing principles. Hence, cytotoxic agents are only partially inactivated and contribute to damage of yet-unperturbed cells. Consequently, a chronic inflammatory process results. In this vicious circle of permanent cell destruction, not only novel cytotoxic elements but also novel alarmins and antigens are liberated from affected cells. In severe cases, very low protection leads to organ failure, sepsis, and septic shock. In this review, the major classes of host-derived cytotoxic agents (reactive species, oxidized heme proteins and free heme, transition metal ions, serine proteases, matrix metalloproteases, and pro-inflammatory peptides), their corresponding protective principles, and resulting implications on the pathogenesis of diseases are highlighted.

## 1. Introduction

Persistent, long-lasting inflammation is a general problem in many diseases. As these chronic inflammatory states are only insufficiently or not terminated, novel immune cells are again and again recruited to and activated at the inflamed loci. Often, an ongoing immunocompromised condition exists in these patients, a condition that further disturbs the recovery to normal tissue homeostasis.

During inflammation, immune responses are initiated by the ligation of pathogen-associated molecular patterns (PAMPs) and/or damage-associated molecular patterns (DAMPs) to pattern-recognition receptors (PRRs) [1,2]. DAMPs are host-derived molecules that are also known as danger signals or alarmins. PRRs are distributed in both cell membranes and cytoplasm [3,4]. As a result of PAMP and DAMP ligation to PRRs, signaling events are induced leading to the release of cytokines and antimicrobial agents and the attraction of immune cells [5]. These activities are generally directed to combat invading pathogens and to decrease tissue damage. A second activity initiated by PRRs concerns the maturation of dendritic cells for the presentation of antigens to T lymphocytes [5].

This very attractive concept of immune activation by molecular patterns covers a broad range of initiating molecules including constituents of pathogens (viruses, bacteria, fungi, and others) and agents from damaged host cells. This concept is closely associated with immune responses initiated by external and host-derived antigens. Important biomarkers of an ongoing inflammation are pro-inflammatory cytokines such as interleukin-1 (IL-1), interleukin-6 (IL-6), and tumor necrosis factor α (TNF-α) and acute-phase proteins such as C-reactive protein (CRP) and serum amyloid A (SAA) [6,7,8,9,10,11]. The termination of inflammation is also highly regulated and characterized in contrast to the initiation and propagation phases by an own set of cytokines and changed energy metabolism of immune cells [10,12]. Importantly, anti-inflammatory cytokines contribute not only to the resolution of immune responses but also to the replacement of damaged biological material by novel cells and components of the extracellular matrix [13,14]. A transient immunosuppression is typical of this phase of inflammation [15]. 

During inflammation, unperturbed cells and tissues of the host can be damaged. Despite huge progress in understanding molecular and regulatory aspects of inflammation, no clear answers are given about the general interplay between inflammation and cell and tissue destruction, the severity of the resulting damage, and the fate of the affected organism during chronic inflammation. 

Besides physical factors such as traumata, heat, or cold impacts, the action of numerous cytotoxic agents can seriously affect intact cells and tissues [16]. Like the initiating factors of inflammation, cytotoxic agents can result from both external sources and affected cells and tissues of the host. In the initiation and propagation phases of inflammation, cytotoxic agents from activated immune cells and defective tissue cells act predominantly destructively. During the resolution of inflammation, a shifted balance in the synthesis of extracellular matrix can also affect tissue homeostasis. 

In this review, the role of host-derived cytotoxic agents will be evaluated in the development of cell and tissue damage during inflammation. In addition, deviations in the balance between cytotoxic agents and protective principles will be highlighted. On this basis, the role of insufficient protection against damage in the development of chronic inflammatory states will be addressed.

## 2. The Balance between the Action of Cytotoxic Agents and Protective Principles

### 2.1. Major Classes of Host-Derived Cytotoxic Agents

**Figure 1:**
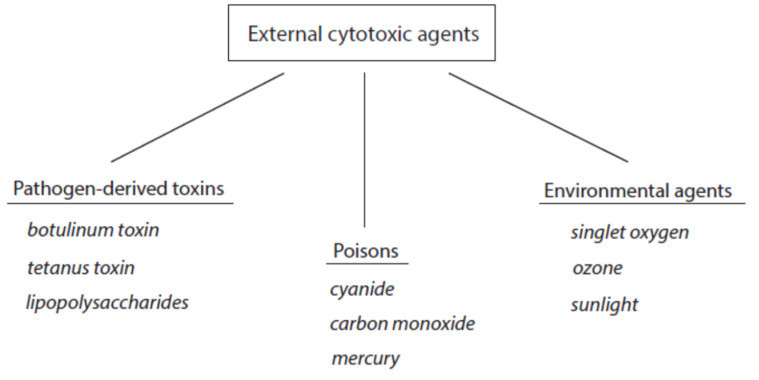
Classification of external cytotoxic agents. Three selected examples are indicated for each group.

The contact of cytotoxic agents with living matter worsens cell functions and can induce irreversible changes in cells and tissues including cell death. According to the source of these agents, they can be roughly divided into external and host-derived cytotoxic components. The group of external cytotoxic agents comprises pathogen-derived toxins and manifold external poisons that act on the organism by inhalation, direct contact, or uptake with food. A third group represents environmental cytotoxic agents (for details see Section 2.4). An overview about external cytotoxic agents is given in Figure 1. Selected examples of these agents are included.

Of course, these external cytotoxic agents can cause sufficient threat to the affected tissues and finally the death of the organism. However, these agents are usually not involved in long-lasting, chronic inflammation.

Host-derived cytotoxic agents result from activated immune cells such as neutrophils, eosinophils, monocytes, macrophages, and T cells, but also from affected tissue cells of non-immunological origin, for instance, muscle cells and red blood cells. Immune cells contain an arsenal of potentially cytotoxic agents that are needed to inactivate and kill pathogens and to remove and digest affected cells and destroyed tissues [10]. Usually these agents act within small, bounded compartments, e.g., within the phagosomes of neutrophils and macrophages. However, a certain amount of these cytotoxic agents is released from activated immune cells into the surrounding milieu, where they become dangerous to unperturbed cells. The following classes of immune-cell-derived cytotoxic agents are known: small reactive species, heme peroxidases, free metal ions, serine proteases, matrix metalloproteases, and small pro-inflammatory peptides. These agents are either pre-assembled or generated during cellular immune activation. 

Damage to tissue cells of non-immunological origin can result in the uncontrolled release of heme proteins such as hemoglobin and myoglobin and the subsequent formation of free heme. Cellular stress is also associated with enhanced formation of reactive species and deviations in free metal ion metabolism. As a result, the metabolism of mitochondria is disturbed and numerous oxidative processes in biological constituents take place.

**Figure 2:**
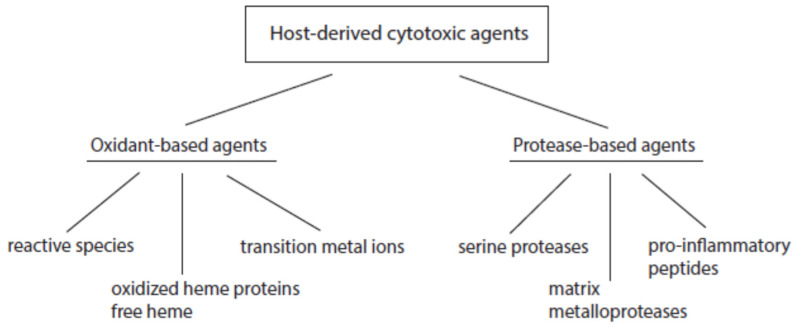
Major classes of host-derived cytotoxic agents.

According to their mode of action, host-derived cytotoxic agents can be divided into oxidant- and protease-based agents (Figure 2). Products of the first group promote oxidative alterations of biological constituents, whereas members of the second group cause proteolytic cleavage in cell and tissue components.

An overview of the major host-derived cytotoxic agents is given in Table 1. This overview also includes key information about the modes of action of these dangerous molecules and naturally occurring protective principles to avoid substantial damage. More details about host-derived cytotoxic agents are given in Section 3.

### 2.2. Control of Cytotoxic Agents by Protective Principles

The destructive action of host-derived cytotoxic agents depends not only on the mass of released cytotoxic agents, but also on the current status of host-own protective principles [10,17]. In order to curtail or avoid destruction by these agents, numerous ready-to-use mechanisms exist in cells and tissues to inactivate immediately hazardous components released from activated immune and affected tissue cells. Usually, unperturbed cells and tissues are well equipped with protective principles. In this way, any threat to unperturbed tissue components is minimized. The major antagonizing principles are listed in Table 1 in relation to their cytotoxic agents.

**Figure 3:**
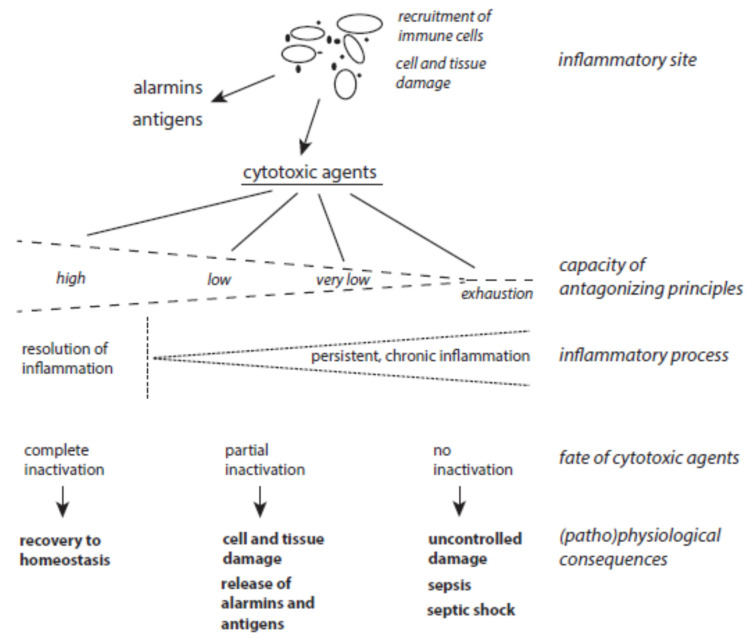
The interplay between host-derived cytotoxic agents and antagonizing principles. (Patho)physiological consequences of the release of cytotoxic agents at inflammatory sites highly depend on the status of protective mechanisms.

The balance between host-derived cytotoxic agents and protective principles functions well as long as the activation of immune cells is moderate enough and neighboring tissues are well-equipped with ready-to-use protective mechanisms (Figure 3). Problems can arise with severe and long-lasting immune responses and with the decline, exhaustion, or inactivation of selected antagonizing principles despite an up-regulation of many protective proteins under stress situations. In turn, long-lasting inflammatory processes can result from the permanent release of cytotoxic agents from damaged cells in combination with insufficient inactivation of these agents. In other words, low expression of a few protective principles favors the continuous action of destructive agents and affects still-unperturbed cells. In this vicious circle of permanent cell destruction, not only novel cytotoxic elements but also novel alarmins and antigens are liberated from affected cells. In addition, pro-inflammatory peptides such as angiotensin II and bradykinin are formed by insufficient inactivation of serine proteases. Hence, the inflammation cannot be terminated sufficiently and flares up again and again. In severe cases, a very low level of protection leads to organ failure, sepsis, and septic shock.

To overcome chronic inflammation, it is highly essential, besides inhibition of selected pathways in the inflammatory cascade, to improve poorly expressed protective systems to better detoxify the damaging agents.

### 2.3. Disturbed Balance between De Novo Synthesis and Damage of Tissue Components during Resolution of Inflammation

Termination of inflammation is characterized by the down-regulation of pro-inflammatory cells, cytokines, and signaling pathways as well as by the formation of anti-inflammatory mediators and induction of repair processes. During this phase of inflammation, cytokines of the transforming growth factor β (TGF-β) family, which are secreted from M2-type macrophages and some other cells, suppress together with interleukin 10 (IL-10)-activated immune cells [15,18]. These cytokines also promote tissue repair by stimulating fibroblasts to synthesize collagen and other components of the extracellular matrix (ECM) and by the release of tissue inhibitors of metalloproteases (TIMPs) [19,20]. The latter inhibitors down-regulate the activity of matrix metalloproteases (MMPs) and thus prevent degradation of ECM components.

### 2.4. Selected Environmental Cytotoxic Agents

Although not host-derived, we can also be exposed to external cytotoxic agents (see Figure 1). Of these agents, environmental cytotoxic agents act more or less intensely and permanently on our organism. As this exposure concerns nearly all persons, these agents are usually detoxified by antagonizing principles when the exposure is moderate and does not exceed a critical level. Examples of environmental cytotoxic agents, their mode of action, and antagonizing principles are given in Table 2. 

## 3. Selected Cytotoxic Agents and Their Counter-Regulating Principles 

### 3.1. Small Reactive Species and Metal Ions

#### 3.1.1. Superoxide Anion Radicals 

The stepwise reduction of dioxygen yields the species superoxide anion radical (O_2_^•−^) and hydrogen peroxide (H_2_O_2_) [36]. These species are less dangerous concerning their direct action on tissue components. However, they are involved in the formation of highly reactive and tissue-damaging agents by interaction with radicals, metal ions, and iron-containing proteins.

Activated leukocytes are able to generate large amounts of O_2_^•−^ by reducing dioxygen. This reaction is catalyzed by NADPH oxidase, which is assembled from several membranous and cytoplasmic components during the activation of neutrophils, eosinophils, monocytes, and macrophages [37,38]. NADPH oxidases are also distributed in cells of the blood vessel wall, respiratory tract, gastrointestinal tract, and thyroid gland [39,40,41]. However, these enzymes are less efficient in reducing dioxygen than NADPH oxidase from immune cells. Other sources for superoxide anion radicals are reactions of xanthine oxidase [42,43], autoxidation of hemoglobin and myoglobin [44,45], cytochrome P450-driven redox recycling of some xenobiotica [46,47], and one-electron reduction of dioxygen by different mitochondrial enzymes [48,49]. 

Superoxide anion radicals are unstable. Two superoxide anion radicals dismutate spontaneously to hydrogen peroxide and dioxygen [50]. The rate of this dismutation highly depends on pH, with a maximal rate around pH 4.8, the pk_a_ value of O_2_^•−^, and decreasing rates with increasing pH [51]. With one unit pH increase, the dismutation rate of O_2_^•−^ decreases by one order of magnitude. At pH 7.4 this rate is 2 × 10^5^ M^−1^s^−1^ [51].

Superoxide anion radical reacts in a very rapid reaction with nitrogen monoxide, also a radical species, under the formation of the powerful oxidant peroxynitrite [52,53]. In mitochondria, superoxide anion radical is able to release Fe^2+^ from molecules containing [4Fe-4S]^2+^ clusters such as aconitase [54,55]. 

In humans, control over O_2_^•−^ is realized with three isoforms of superoxide dismutase (SOD) and cytochrome c. SOD1 is distributed in the cytoplasm, intermembrane space of mitochondria, and nuclei [56,57]. In the mitochondrial matrix, SOD2 dominates [58]. SOD3 is mostly found in blood vessel walls and lungs [59]. These enzymes catalyze the dismutation of O_2_^•−^ with a rate several orders higher than the spontaneous dismutation reaction of O_2_^•−^. In the intermembrane space of mitochondria, oxidized cytochrome c oxidizes O_2_^•−^ to O_2_, thus contributing to the detoxification of O_2_^•−^ [60,61].

**Figure 4:**
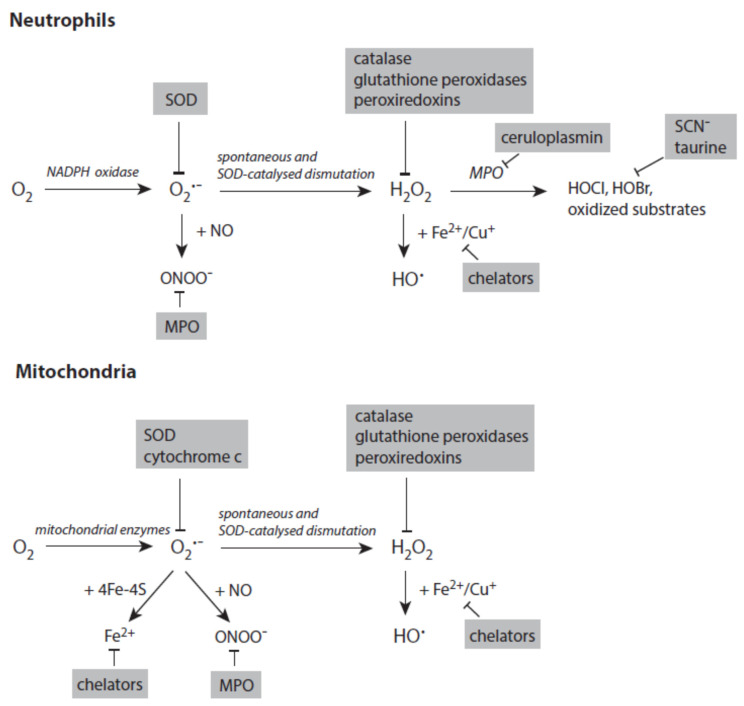
Major pathways in formation of reactive species in activated neutrophils (**upper panel**) and stressed mitochondria (**lower panel**). Antagonizing principles against these species are displayed on grey backgrounds. In deactivation of transition metal ions, the term chelators stands for numerous proteins that scavenge, transport, and store iron and copper ions. Further explanations are given in the text. Abbreviations: MPO—myeloperoxidase, SOD—superoxide dismutase.

Figure 4 depicts the major pathways for the formation of reactive species with a special focus on processes in activated neutrophils and stressed mitochondria. In both systems, the generation of small reactive species starts with the reduction of dioxygen to superoxide anion radicals. 

#### 3.1.2. Hydrogen Peroxide

Spontaneous and SOD-catalyzed dismutation of O_2_^•−^ represent the main route of formation of H_2_O_2_. Thus, all processes generating O_2_^•−^ also yield H_2_O_2_. Otherwise, different peroxisomal enzymes are able to reduce O_2_ directly to H_2_O_2_ [62]. 

Due to its electronic structure, reactions of H_2_O_2_ are restricted to transition metal ions, complexes of these ions, and some proteins with selenocysteine (or cysteine) residues at the active site [63,64]. Hydrogen peroxide is freely permeable through biological membranes, unlike O_2_^•−^. The interaction of transition metal ions such as Fe^2+^ and Cu^+^ with H_2_O_2_ yields very reactive hydroxyl radicals and metal-based reactive species that can cause manifold damaging reactions on biological material [65,66]. 

Heme peroxidases, different cytochromes, hemoglobin, and myoglobin are activated by H_2_O_2_ leading to reactive states of the heme in these proteins. During immune response, H_2_O_2_ activates the heme peroxidases myeloperoxidase (MPO), eosinophil peroxidase (EPO), and lactoperoxidase (LPO), which are involved in both pro- and anti-inflammatory activities [17,67,68,69,70].

Several enzymes are known to catalyze the reduction of H_2_O_2_ to H_2_O (Figure 4). Glutathione peroxidase (GPX) utilizes glutathione (GSH) to reduce H_2_O_2_. The highly distributed isoforms GPX1 and especially GPX4 also detoxify peroxynitrite, lipid hydroperoxides, and other organic hydroperoxides [71,72]. GSH is recovered from the resulting oxidized glutathione (GSSG) by glutathione reductase [73]. Peroxiredoxins, which are closely coupled to the thioredoxin system, also efficiently reduce H_2_O_2_ to H_2_O [74]. Catalase removes H_2_O_2_ by both reduction to H_2_O and oxidation to O_2_ [75].

#### 3.1.3. Transition Metal Ions and Hydroxyl Radicals

In the reaction between H_2_O_2_ and Fe^2+^, which is known as the Fenton reaction, the highly reactive hydroxyl radical is formed. Alternatively, iron–oxygen complexes such as ferryl or perferryl compounds are discussed as products of this reaction [65,66]. Similarly, the reaction of H_2_O_2_ with Cu^+^ also yields hydroxyl radicals [76]. Organic hydroperoxides are also oxidized by Fe^2+^ and Cu^+^ under the formation of reactive radical species that are involved in subsequent destructive reactions. Beyond Fenton chemistry, further mechanisms apparently contribute to metal-ion-induced tissue damage such as the interaction of Fe^2+^ with biological buffer components or the formation of Fe^2+^–O_2_ and Fe^2+^–O_2_–Fe^3+^ complexes [77,78,79,80,81].

Hydroxyl radicals react in a nearly diffusion-controlled manner with many substrates by abstraction of an H-atom or by addition to an unsaturated system under formation of a hydroxylated product [82]. In both reaction types, substrate radicals are formed that can undergo manifold further reactions.

To avoid the disastrous formation of reactive species such as hydroxyl radicals and others, the main strategy of living matter is the tight control of transport, storage, and utilization of free metal ions (Figure 4) as both iron and copper ions are necessary constituents of many proteins [83,84]. Major components controlling iron metabolism are hepcidin (intestinal absorption), transferrin (blood transport), transferrin receptor (uptake by cells), and ferritin (intracellular storage) [85,86,87,88]. Similarly, different import and export transporters and chaperones are involved in copper metabolism [89]. Ceruloplasmin is able to oxidize both Fe^2+^ and Cu^+^ [90]. Lactoferrin released from activated neutrophils binds Fe^3+^ and promotes its transfer to transferrin [91].

#### 3.1.4. Peroxynitrite

As already mentioned, peroxynitrite is formed in a very rapid reaction between O_2_^•−^ and NO [52,53]. Peroxynitrite is involved in the formation of thiyl radicals and nitration of tyrosine residues, and is able to induce lipid-peroxidation processes [92,93,94,95]. In reaction with CO_2_, it yields nitrosoperoxycarbonate, which can decompose into radical species [96,97]. 

At inflammatory sites where heme peroxidases are present, peroxynitrite is decomposed in its reaction with resting MPO [98,99,100]. Other redox-active heme proteins scavenge peroxynitrite and inactivate this powerful oxidant [101,102,103].

#### 3.1.5. Heme Peroxidases and Hypohalous Acids

At an inflammatory site, the heme protein MPO can be released from activated neutrophils (Figure 4) [67,104]. Eosinophils contain a similar peroxidase, the eosinophil peroxidase (EPO) [105]. A third immunologically relevant heme peroxidase is LPO, which is distributed in mucous surfaces [68]. All three heme peroxidases are able to oxidize SCN^−^ to ^−^OSCN. MPO and EPO also oxidize Br^−^ to HOBr, whereas only MPO is able to yield HOCl from Cl^−^ oxidation [70].

The MPO product HOCl reacts efficiently with methionine and cysteine residues of proteins. Further major protein targets for HOCl are residues of cystine, histidine, tryptophan, lysine, and α-amino groups [106,107]. HOBr , like HOCl, also oxidizes many residues in proteins, especially cysteine and methionine ones. HOBr induces ring halogenation in tyrosine residues more efficiently than HOCl [108].

Both HOCl and HOBr are inactivated at a high rate by thiocyanate (SCN^−^) [109,110]. HOCl is additionally inactivated by Br^−^. Further antagonizing principles against both hypohalous acids are ascorbate, GSH, taurine, and, additionally for HOBr, urate [111]. 

In blood, MPO and EPO are inactivated by ceruloplasmin through the formation of a tight inhibitory complex between heme peroxidase and ceruloplasmin [112,113,114,115]. 

### 3.2. Hemoglobin and Myoglobin Metabolites

**Figure 5:**
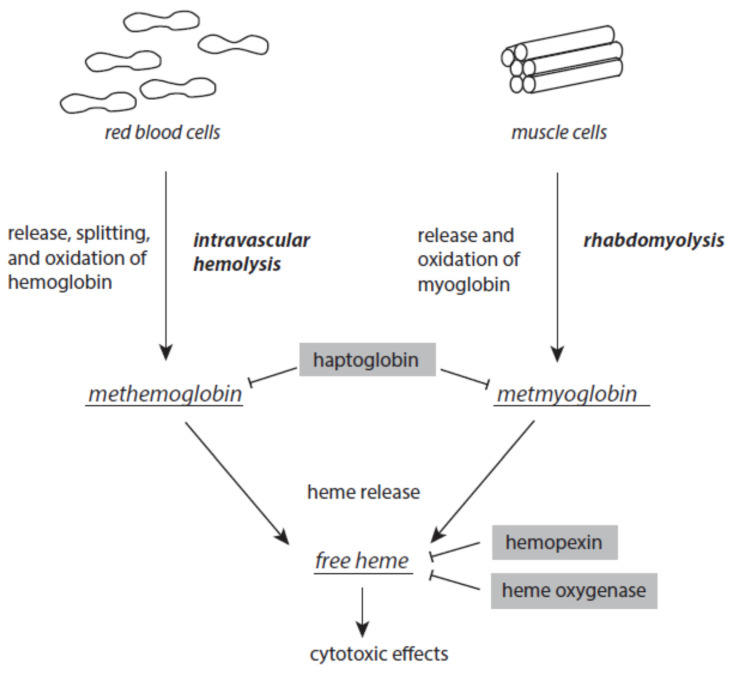
Formation of methemoglobin, metmyoglobin, and free heme as a result of excessive intravascular hemolysis and rhabdomyolysis. Protective mechanism are presented on grey backgrounds. Further explanations are given in the text.

There is always a release of intact hemoglobin from red blood cells and myoglobin from muscles at low levels. Intravascular hemolysis and rhabdomyolysis can be markedly enhanced under stress and disease situations (Figure 5). Once released from red blood cells, tetrameric ferric hemoglobin dissociates into dimers and is easily oxidized to methemoglobin. This oxidation is usually caused by nitric monoxide. Excessive intravascular hemolysis can affect the bioavailability of NO [116,117]. The serum protein haptoglobin is able to scavenge free methemoglobin. The resulting haptoglobin–methemoglobin complex is eliminated from circulating blood by spleen and liver macrophages in a CD163-dependent process [118,119]. In a similar way, haptoglobin also scavenges metmyoglobin formed after the release of myoglobin from muscle cells. 

Although it is an acute-phase protein, the capacity of haptoglobin is limited when severe intravascular hemolysis or rhabdomyolysis occur. Both methemoglobin and metmyoglobin spontaneously liberate ferric protoporphyrin IX, briefly known as free heme, a very dangerous molecule [120]. Free heme easily intercalates into the lipid phases of membranes and lipoproteins and the hydrophobic areas of proteins. At these loci, it catalyzes oxidative processes [121,122]. In intact red blood cells, free heme induces hemolytic processes, thus enhancing existing intravascular hemolysis [123,124]. Free heme is highly cytotoxic to kidney and liver [125,126]. It is also a ligand to toll-like receptor 4 and thus contributes to the intensification of inflammatory processes [127,128]. In the nucleus, free heme interacts with parallel guanine-rich quadruplex DNA and RNA structural elements, known as G4 structures [129,130].

In order to avoid the disastrous activities of free heme, different serum proteins are able to complex and inactivate free heme. Hemopexin binds free heme with high affinity. This free-heme–hemopexin complex is liberated from circulating blood via CD91-mediated internalization by hepatocytes [131]. In humans, unlike mice, hemopexin is not an acute-phase protein [132].

Inside cells, free heme is detoxified by an interaction with heme oxygenase [133,134].

### 3.3. Oxidation of Cell and Tissue Components

In addition to proteolytic cleavage, lipids, proteins, nucleic acids, and carbohydrates are subjected under stress conditions to numerous chemical processes, whereby oxidative modifications predominate [135]. The major initiating agents of these oxidative processes are highly reactive species, free transition metal ions, free heme, and aldehydes. Besides the open chain form of glucose [136], aldehydes result mostly from oxidative modifications of lipids [137,138].

Oxidative alterations of biological substrates are counterbalanced by lipid- and water-based antioxidant mechanisms. In lipid phases, major natural antioxidants are α-tocopherol, β-carotene, ubiquinol, and dehydrolipoic acid. They are mainly involved in the scavenging of lipid peroxyl radicals [139,140,141]. Inactivation of lipid hydroperoxides is a further strategy to prevent oxidative processes. This is achieved most of all by the action of glutathione peroxidase 4 (GPX4). A high intracellular level of GSH is essential for the proper action of GPX4 and other glutathione peroxidases [71,72]. In addition, perturbed acyl residues in phospholipids are cleaved by phospholipases [142]. A thorough control over transition free metal ions also contributes to the prevention of oxidative processes in membranes and lipoproteins.

Urate and ascorbate are the main water-soluble antioxidants in our organism [143,144]. Different polyphenols are important dietary antioxidants [145]. They exert their protective action by radical scavenging, sequestration of free metal ions, and interaction with activated complexes of heme proteins [146,147,148].

### 3.4. Serine Proteases

#### 3.4.1. Release of Serine Proteases from Immune Cells

At inflammatory sites, activated neutrophils can release the serine proteases elastase, cathepsin G, proteinase 3, and neutrophil serine protease 4. These proteases are primarily involved in the deactivation, killing, and digestion of phagocytosed microorganisms in neutrophils. Their pH optimum is around 8–9, a condition that predominates in early phagosomes of neutrophils [149,150]. Elastase exhibits a killing activity against Gram-negative bacteria [151,152] and a variety of cancer cells [153]. In cancer cells, unlike non-cancer cells, elastase cleaves CD95 to liberate a death domain fragment that acts cytotoxically together with histone H1 [153]. 

Serine proteases participate in the recruitment of neutrophils to a destination site by digestion of the surrounding tissue components and the induction and regulation of immune signaling. Elastase and proteinase 3 are able to cleave a broad range of chemokines and cytokines [154]. The substrate specificity of cathepsin G is also relatively broad but more restricted than that observed for elastase and proteinase 3 [155]. In these experiments, only a few cytokines and chemokines, such as TNF-α, interleukin 5 (IL-5), interleukin 8 (IL-8), macrophage colony-stimulating factor (M-CSF), monocyte chemoattractant protein 1 (MCP-1), IL-1α, and Rantes, were resistant to neutrophil serine proteases. 

Elastase and other serine proteases are attached together with other neutrophil proteins to a DNA network in neutrophil extracellular traps. These traps can kill external microbes independent of phagocytosis [156,157].

Activated mast cells release the serine proteases chymase, tryptase, and cathepsin G [158]. These proteases are involved in matrix destruction, tissue remodeling, and regulation of inflammation. Mast cell tryptase and chymase are more restrictive than neutrophil serine proteases in the cleavage of chemokines and cytokines [154,155].

#### 3.4.2. Activities of Neutrophil Serine Proteases

**Figure 6:**
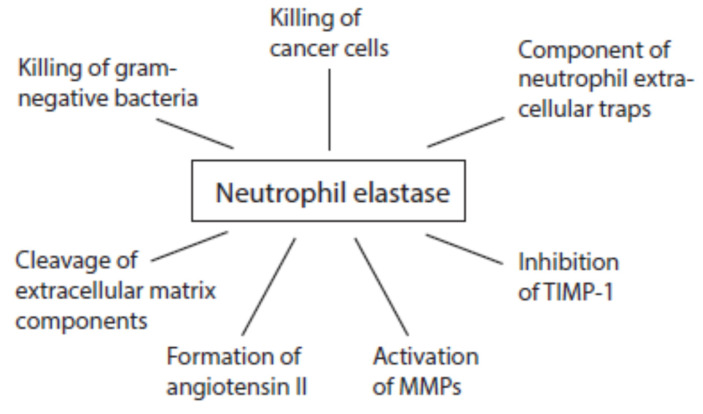
Activities of neutrophil elastase at inflammatory sites.

Although all serine proteases contribute to damaging reactions, the focus is mostly directed on elastase. An overview about multiple activities of neutrophil elastase during immune response is given in Figure 6. Once released from activated neutrophils, elastase can affect healthy tissues. Elastase is involved in the destruction of extracellular matrix components such as elastin, collagens, proteoglycans, and laminin [159]. 

Like cathepsin G, proteinase 3, and cathepsin B, neutrophil elastase is able to convert angiotensinogen and angiotensin I into angiotensin II [160,161,162]. This pro-inflammatory peptide can further foment inflammatory processes.

At inflammatory sites, neutrophil elastase activates MMP2, MMP3, and MMP9 from inactive precursors by cleaving an inhibitory protein residue [163,164,165,166]. Cathepsin G is also able to activate MMP3 [163]. Cathepsin G and proteinase 3 are involved in MMP2 activation [164]. Elastase might additionally degrade TIMP-1 [165,167]. 

#### 3.4.3. Mast Cell Serine Proteases

Human mast cells contain several types of tryptases and two members of chymase-like proteins, namely α-chymase and cathepsin G, which are secreted in response to allergens and pathogens [158]. Mast cell proteases are known to stimulate the production of pro-inflammatory mediators such as IL-6 and IL-8 from bronchial epithelial cells and promote procollagen cleavage. With these activities they contribute to the recruitment of neutrophils and eosinophils at inflamed epithelium [168,169,170,171]. 

Other inflammation-promoting activities of chymase are the cleavage of angiotensin I into angiotensin II, activation of MMPs, and release of selected extracellular matrix elements [172]. Tryptase is involved in the degradation of fibronectin and chemokines [172]. Both tryptases and chymases contribute to the activation of different MMPs [173,174,175]. MMPs are implicated in the pathogenesis of atherosclerosis and abdominal aortic aneurysms [176,177,178,179,180]. Mast cell proteases are implied in airway epithelial remodeling and alterations in epithelium functions [181]. They also contribute to angiogenesis induction during tumor growth [182]. Chymase promotes the formation of active TGF-β from its precursor [183].

Tetrameric tryptase is stabilized by heparin and some other glycosoaminoglycans [184]. In this complex, tryptase is not accessible to anti-proteases such as A1AT, SPLI, and α_2_-macroglobulin [185,186]. Lactoferrin, myeloperoxidase, and antithrombin III, which are known to have heparin-binding domains, can inhibit tryptase activity [187,188,189,190]. Spontaneous dissociation of the tryptase tetramer is a further mechanism to control tryptase activity [184,191].

#### 3.4.4. Antiproteases

**Figure 7:**
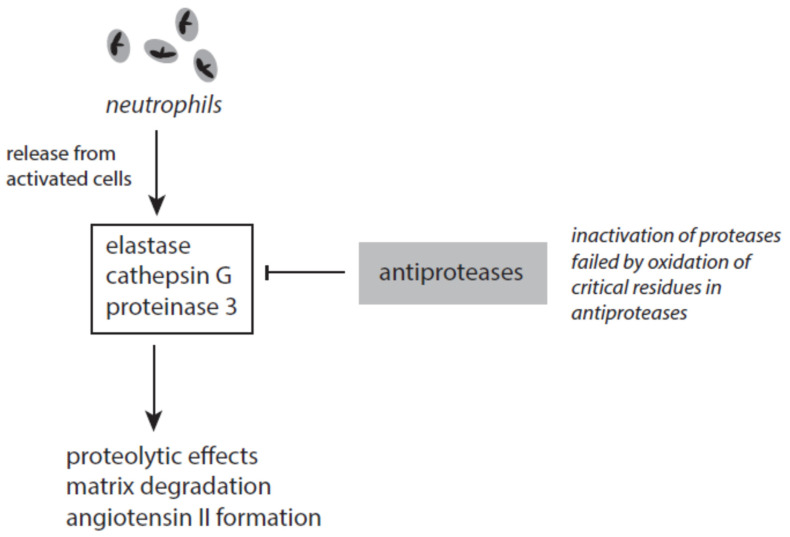
The interplay between neutrophil-derived serine proteases and antiproteases.

Several antagonizing proteins against elastase and other serine proteases exist in blood and tissues (Figure 7). The most abundant anti-protease is the serpin α_1_-antitrypsin (A1AT). This serum protein is synthesized in the liver and represents an acute-phase protein. A1AT inhibits elastase and cathepsin G but not in the presence of heparin [192,193]. The activity of proteinase 3 is affected by A1AT to a lesser degree. Heparin, however, enhanced the inactivation of proteinase 3 [194].

Several factors contribute to the failure of A1AT to inhibit elastase. The inactivation of elastase requires two unperturbed methionine residues (Met-351 and Met-358) at the active site of A1AT. By oxidation of these residues A1AT loses its ability to inhibit elastase [195]. Under stress conditions methionine oxidation in A1AT can be initiated by highly reactive species such as hydroxyl radicals, peroxynitrite, HOCl, HOBr, and others [196]. Tobacco smoke and activated phagocytes are under discussion to contribute to methionine oxidation in A1AT and thus cause an acquired A1AT deficiency [196]. Furthermore, neutrophil elastase can bind to negatively charged surfaces and polymers. Surface-bound elastase cannot be inhibited by endogenous antiproteases [197]. 

Serpin A3, also known as α_1_-antichymotrypsin, is, like A1AT, an acute-phase protein. This antiprotease efficiently inactivates cathepsin G and mast cell chymase [198,199,200]. 

Secretory leukocyte protease inhibitor (SLPI) is able to inactivate several serine proteases such as neutrophil elastase, cathepsin G, tryptase, and chymase [201]. SLPI is constitutively expressed in mucous secretions [202,203] and also secreted from activated immune cells. It is assumed that SLPI exhibits an anti-apoptotic effect on immune cells and thus contributes to a better removal of dying cells and microbes at inflammatory sites [204]. 

Elafin, which is also known as proteinase inhibitor 3, is able to inactivate neutrophil elastase and proteinase 3 [205,206]. It exerts anti-inflammatory, anti-microbial, and wound-healing effects [205,206]. Contradictory results were reported about the action of elafin on tumorigenesis. These results range from promotion of cell proliferation and induction of resistance against chemotherapy to tumor-suppressive effects [207,208]. In early-stage hepatocellular carcinoma, elafin promotes metastasis formation via activation of EGFR/AKT signaling [209].

The antiprotease serpin B1 efficiently inactivates elastase, cathepsin G, and proteinase 3 [210]. Under oxidative stress, the cysteine residue at the active site in serpin B1 is oxidized with the loss of the antiprotease activity.

In contrast to the aforementioned antiproteases, which directly interact with the active site of proteases, α_2_-macroglobulin forms a tetrameric cage around active proteases, thus inhibiting the direct contact between protease and substrate molecules. In this way, large substrate molecules such as collagen are excluded from direct contact, whereas small peptide substrates can be digested [211,212]. Although α_2_-macroglobulin inhibits the activities of elastase, cathepsin G, proteinase 3, and MMP9 released from neutrophils [213,214,215], the complex between elastase and α_2_-macroglobulin is still active against small substrates [214]. Moreover, neutrophil-derived reactive species such as HOCl can hinder α_2_-macroglobulin to form tetramers and promote stabilization of dimers with the loss of the antiprotease activity [216,217].

High-affinity complexes are also known between ceruloplasmin and serine proteases of neutrophils [91]. In this way, a destructive action of serine proteases on tissue components is minimized. 

### 3.5. Small Pro-Inflammatory Peptides

#### 3.5.1. Angiotensin II

The peptide hormone angiotensin II is an essential part of the renin–angiotensin–aldosteron system. It is involved in the regulation of blood pressure and water metabolism. During this activity, angiotensin II is formed from angiotensin I by the angiotensin-converting enzyme (ACE).

**Figure 8:**
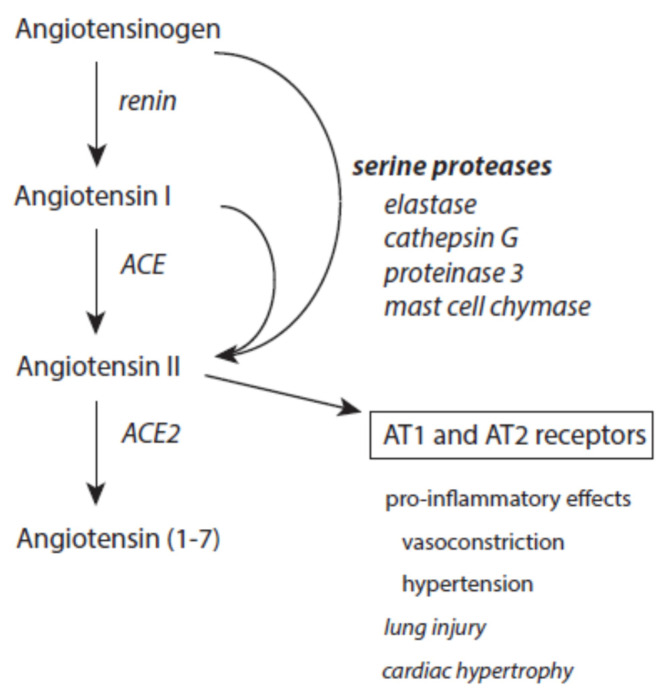
Effects of serine proteases on the renin–angiotensin–aldosteron system. Further explanations are given in the text.

At inflammatory sites, angiotensin II can also be produced from cleavage of both angiotensinogen and angiotensin I by serine proteases released from immune cells such as elastase, cathepsin G, proteinase 3, and mast cell chymase (Figure 8) [160,161,162,218]. Increased angiotensin II contributes via docking to AT1 and AT2 receptors to proteolysis, actin cleavage, apoptosis induction, and activation of the ubiquitin-mediated protein degradation [219,220,221]. It also promotes superoxide anion radical production via activation of NADH/NADPH oxidases [222]. Generally, these pro-inflammatory activities of angiotensin II mediate the prolonged existence of inflammatory states [223].

Angiotensin II is under the control of ACE2, which converts this octapeptide to angiotensin 1–7 [224]. This limits the devastating activity of angiotensin II. Moreover, angiotensin 1–7 exerts an anti-inflammatory activity [225]. 

#### 3.5.2. Bradykinin

As an essential member of the contact system, the nonapeptide bradykinin is responsible for increased vascular permeability, vasodilation, hypotension, and other effects via interaction with its constitutively expressed B_2_ receptor [226,227]. A further pro-inflammatory metabolite is des-Arg9-bradykinin, which is formed from bradykinin by carboxypeptidase N. At inflammatory sites, des-Arg9-bradykinin acts selectively via bradykinin B_1_ receptors, which are only expressed in inflamed and injured tissue [228,229,230].

Bradykinin is a short-lived mediator of inflammation. It is inactivated by aminopeptidase P and the angiotensin-converting enzyme (ACE). Inhibition of ACE enhances bradykinin’s effects [229].

### 3.6. Inhibition of Matrix Metalloproteases

In human tissues, 23 MMPs and four TIMPs are found. Most MMPs are normally not expressed in healthy tissue. The activity of MMPs is essential in tissue remodeling, such as angiogenesis, bone growth, wound healing, and repair processes during the resolution of inflammation [231,232]. 

MMPs are secreted as inactive enzymes bearing an inhibitory prodomain that must be cleaved. In addition to neutrophil serine proteases (see Section 3.4.2), plasmin, chymases, and other MMPs are involved in MMP activation [233]. At low concentrations, highly reactive species such as HOCl, ^•^OH, and ONOO^−^ can activate MMPs. However, higher concentrations of these species inactivate active MMPs [234]. 

During the exudation and infiltration phase of inflammation, MMP2 and MMP9 are mainly secreted from invading immune cells, smooth muscle cells, and fibroblasts [235,236,237]. These and other MMPs contribute to cleaving the matrix components collagen and elastin.

The activity of MMPs is tightly controlled by TIMPs and α_2_-macroglobulin. The latter inhibitor, which has a very broad activity range against proteases, acts in blood and other biological fluids [238]. Generally, TIMPs have a broad spectrum of inhibition of MMPs. The constitutively expressed TIMP-2, like TIMP-3 and TIMP-4, is able to inhibit nearly all MMPs. TIMP-1 has a low activity against membrane-bound MMPs [232]. TIMP-3 additionally inhibits members of disintegrin metalloproteinases. Moreover, it is the only TIMP that binds to the ECM [239]. 

## 4. Enhanced Cell and Tissue Damage during Chronic Inflammatory Diseases 

### 4.1. Most Prominent Degradative Agents

Of note, most aforementioned host-derived cytotoxic agents execute a dual role in cells and tissues. They are involved in numerous beneficial functions during metabolism and immune response. Thus, these agents are mandatory to ensure tissue homeostasis and normal functioning of the organism. To control their bad side numerous protective mechanisms help to minimize the destruction of biological constituents. 

Despite the long list of host-derived damaging agents and counter-regulating principles, only a few of these agents are responsible for initiating cell and tissue degradation under pathological conditions. The reason for this damage is mainly the weakness or exhaustion of the corresponding protective system. In turn, this favors prolonged activity of the damaging agents, induces the release of DAMPs and antigens from perturbed cells and tissues, and causes attraction of further immune cells. 

Considering the aforementioned data, the most prominent candidates for this failure are the loss of control over the sequestration of transition metal ions, exhaustion of haptoglobin and hemopexin, enhanced activity of elastase, the disastrous action of angiotensin II, and the disturbed balance between MMPs and TIMPs.

### 4.2. Diminished Control over Transition Metal Ions

Ferrous and cupric ions are several-fold involved in damaging reactions of biological constituents. In stressed mitochondria, enhanced formation of O_2_^•−^ favors the release of Fe^2+^ from proteins with [4Fe-4S]^2+^ clusters and thus contributes to mitochondrial dysfunction and apoptosis induction [240,241]. Stress situations are also responsible for the increase in iron ions in biological fluids and cytoplasm [242,243]. These free ions can result from damaged biological material, necrotic cells, heme destruction, release from ferritin, release from the labile iron pool, and overload of protective systems with transition metal ions. 

In the reduced state, transition metal ions catalyze the oxidation of hydrogen peroxide and organic hydroperoxides. As a result of these reactions, highly reactive hydroxyl radicals and different substrate radicals are generated [65,66]. In oxidized lipids, alkoxyl radical species are formed from lipid hydroperoxides by Fe^2+^. The latter reaction promotes further destructive actions in lipid phases [26,244,245].

Ferroptosis is a special form of programmed cell death that is caused by enhanced values of free iron ions and lipid hydroperoxides [246,247]. In addition to an increased concentration of free iron ions, ferroptosis is promoted by a disturbance in glutathione supply and diminished activity of GPX4. Glutathione is the cofactor for GPX4, which is able to remove lipid hydroperoxides within biological membranes [248,249].

Excessive accumulation of copper ions takes place in patients with Morbus Wilson. This condition is associated with destructive reactions in liver, brain, and other organs initiated by the interaction of copper ions with hydroperoxides [250]. Enhanced values of free copper not bound to ceruloplasmin apparently contribute to the pathogenesis of Alzheimer’s disease [251,252]. 

Highly reactive hydroxyl radicals can also be generated as a result of water radiolysis induced by X-ray or radioactive irradiation [33,253]. 

### 4.3. Haptoglobin and Hemopexin Exhaustion

Exhaustion of haptoglobin and hemopexin promotes the disastrous action of free heme. Both severe intravascular hemolysis of red blood cells and rhabdomyolysis of muscle cells contribute to a decline in these protective proteins.

Enhanced intravascular hemolysis is reported for several diseases such as thalassemia, glucose-6-phosphate dehydrogenase deficiency, malaria, paroxysmal nocturnal hemoglobinuria, hereditary spherocytosis, and some others [117,254,255,256]. Increased hemoglobin release from red blood cells is also favored by osmotic stress, sheer stress, lytic poisons, secreted components from Gram-positive bacteria, chirurgical actions on the cardiovascular system, autoantibodies, oxidative processes in membranes of red blood cells, burn-associated necrosis, hemorrhagic conditions, and storage of blood for transfusion [40,117,119,126]. Increased rhabdomyolysis is observed after intensive muscle exercise, traumata, alcohol and drug abuses, the use of certain medications, electrical injury, heat stroke, prolonged immobilization, and as a result of some infections [257,258].

Decline of plasma haptoglobin is regarded as a marker of intravascular hemolysis [259,260]. In hemolytic diseases, a decrease in hemopexin levels follows haptoglobin depletion [261].

A massive release of hemoglobin from red blood cells, e. g. during malaria [262], or myoglobin from traumatic muscles [263,264] can induce acute kidney injury by several mechanisms. Although tubular heme oxygenase is able to detoxify some amount of free hemoglobin and free myoglobin, this enzyme exerts pro-oxidative and damage-promoting activities at a higher load of these heme proteins [265]. Further damage of tubular cells results from free heme by inducing proteasome inhibition, accumulation of misfolded proteins, and favoring the unfolded protein response [133,134,266,267,268].

### 4.4. Inactivation of Antiproteases

At inflammatory sites, elastase and other serine proteases are released from activated neutrophils. Although different antiproteases limit the activity of elastase by the formation of inactive complexes, elastase can promote long-lasting degradation of extracellular matrix components under conditions of oxidative stress. The latter situation favors the oxidation of critical residues in antiproteases with inactivation of these proteins.

The disastrous action of elastase and the failure of antagonizing principles are discussed in chronic obstructive pulmonary disease (COPD) and other lung diseases [269,270,271,272,273,274,275]. In different lung diseases, elastase affects mucus production and causes mucus hyperplasia [276]. Importantly, the activity of surface-bound neutrophil elastase correlates with parameters of diminished airflow and hyperinflation in lungs [197]. A diminished level of SLPI favors the development of emphysema and fibrosis in the lung [277]. Cathepsin G also plays a role in the pathogenesis of COPD and cystic fibrosis [278].

In hereditary A1AT deficiency, the circulating level of A1AT is decreased and represents a risk factor for the development of COPD and emphysema [279]. This hereditary deficiency of A1AT also promotes fibrosis in liver tissue and the formation of liver cirrhosis.

### 4.5. Disastrous Action of Angiotensin II

A shifted balance between ACE and ACE2 towards ACE promotes angiotensin II effects on the cardiovascular system such as vasoconstriction, hypertension, and cardiac hypertrophy [280]. Angiotensin II is involved in damage to the respiratory system and contributes to acute lung injury and acute respiratory distress syndrome [280]. 

Importantly, ACE2 is the receptor for severe acute respiratory syndrome (SARS) viruses [281]. Upon infection with SARS virus, the expression of ACE2 receptors is markedly downregulated in lungs [282]. The inhibition of ACE2 by SARS-COVID-19 also markedly prolongs the fatal action of angiotensin II on lung tissues [283,284]. In COVID-19 patients, antibodies against angiotensin II were found [285]. Downregulation of ACE in COVID-19 also affects bradykinin metabolism and elevates bradykinin level [286]. 

### 4.6. Disturbed Balance between MMPs and TIMPs

At inflammatory sites, MMPs promote the cleavage of collagen and elastin and can thus impair the stability of blood vessel walls. Hence, instable atherosclerotic plaques, thrombotic events, and aortic aneurysms can result [287,288]. In the formation of aneurysms, inflammatory cells infiltrate into an injured vessel wall. Different MMPs, especially MMP2 and MMP9, contribute together with reactive species, neutrophil elastase, and angiotensin II to the pathogenesis of aneurysms [289,290,291,292]. An increased ratio of MMP to TIMP expression was found in aneurysmal aortic specimens [293,294]. 

In repair processes during the termination of inflammation, the right balance between TIMPs and MMPs is highly important for recovery to normal tissue homeostasis [295,296,297]. Excess accumulation of ECM constituents leads to scar formation and the development of fibrosis in many organs. The major constituent of scars is collagen. Organ fibrosis is often followed by organ failure [298,299]. Generally, these deviations are caused by a limited activity of MMPs and prolonged activation by TGF-ß cytokines. Several pathophysiological factors are discussed to contribute to fibrosis, namely conserved PAMPs from pathogens [300,301], uncontrolled TGF-β signaling [302], T-cell derived cytokines [303], autoantibodies [304], and the action of angiotensin II [305,306].

**Figure 9:**
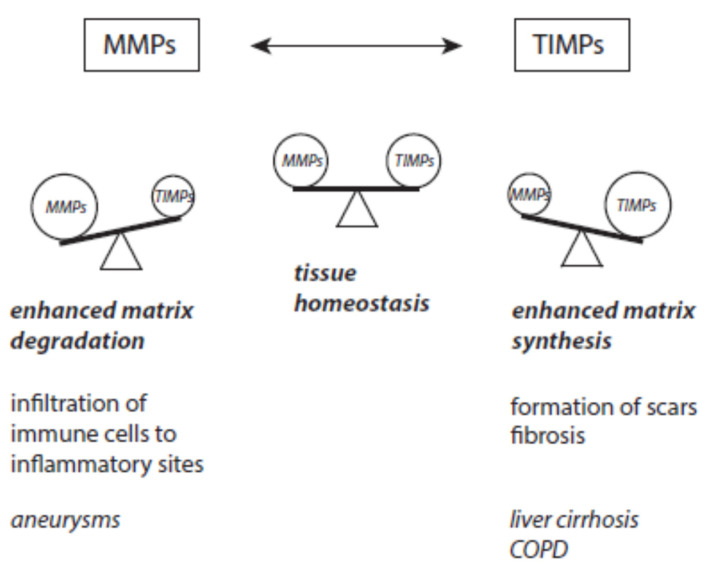
Disturbances of the equilibrium between MMPs and TIMPs and examples of resulting disorders.

The pathophysiological consequences of a disturbed equilibrium between MMPs and TIMPs are schematically presented in Figure 9.

### 4.7. Cytotoxic Agents in Tumorigenesis

In order to survive, many types of tumors manipulate their microenvironment in such a way that immune cells are unable to eliminate these degenerated cells. For example, tumor-associated macrophages are driven by lactate accumulated in cancer cells into an anti-inflammatory M2 subtype [306]. Lactate and extracellular acidosis suppress antitumor immunity and promote angiogenesis and tumor progression [307,308]. Lactate-induced release of hyaluronan by adjacent fibroblasts supports tumor growth, invasion, and metastasis [309]. Moreover, tumor cells can secrete high levels of TGF-β, which dampens the activity of many types of immune cells [310,311]. 

Observations of tumor patients and experimental animals confirm the enhanced formation and release of cytotoxic agents in affected tissue regions. Dysfunctional mitochondria are described in many types of cancers [312]. Oxidative stress can promote the release of iron from 4Fe-4S clusters of mitochondrial proteins such as aconitase and can produce agents damaging mitochondrial DNA [311]. In mice with Lewis lung carcinoma, functional degeneration of mitochondria already occurs at an early disease state [313,314]. 

In patients with pancreatic cancer and other cancer types, the circulating level of TIMP-1 is up-regulated and associated with a poor clinical outcome [315,316]. TIMP-1 activates via binding to CD63 hepatic stellate cells and thus creates a pre-metastatic niche in the liver [317].

Intratumoral hemorrhages and hemoglobin level in tumors are also associated with a poor clinical prognosis for affected individuals [318,319,320]. In these hemorrhages, hemoglobin can be released from red blood cells and the formation of free heme is likely. Indeed, free heme contributes to the progression of prostate cancer by controlling the expression of key target genes via docking to guanine-rich (G4) elements [321]. In the blood of prostate cancer patients, an inverse correlation between the levels of free heme and hemopexin was observed [321].

Elastase released from human neutrophils efficiently kills a wide range of cancer cells in contrast to elastase from murine neutrophils [153]. In neutrophils of mice, unlike human neutrophils, elastase is co-released with the antiprotease SLPI, which dampens elastase’s effects [153]. In the tumor microenvironment, the ability of elastase to kill cancer cells can be abrogated by the release of serine protease inhibitors and suppressing effects on immune cells [322,323].

Otherwise, neutrophil elastase is able to drive tumorigenesis as shown in preclinical studies using elastase knockout mice and pharmacological inhibition [324]. Intriguingly, the incubation time of cancer cells with elastase affects the functional results. While short incubation (1 h) promotes cellular proliferation, prolonged treatment (6–24 h) induces death of cancer cells [325,326].

In 4T1 and CT26 syngeneic mouse tumor models, angiotensin II promotes the formation of an immunosuppressive microenvironment [327]. Overexpression of the AT1 receptor in tumors is associated with more aggressive tumor features and a poor prognosis [328,329].

### 4.8. Diminished Protection during Sepsis

Sepsis and septic shock are very dangerous clinical conditions that can be associated with multiple organ failure and lethal outcome. Immunocompromised persons such as injured, diseased, and elderly people and newborns are most frequently prone to the development of sepsis. On the basis of impaired host immunity, opportunistic microbes, fungi, and latent viruses can be activated during sepsis [330,331,332,333,334,335,336]. These pathogens further deteriorate the health status of patients. Thus, in sepsis, host-derived cytotoxic agents can contribute together with pathogen-derived cytotoxic agents to cell and tissue damage.

Concerning tissue damage in septic patients, no consensus exists about the predominating damaging mechanisms as the actual status of individual protective systems can vary widely from patient to patient [337,338]. Numerous reports exist about the accumulation of immune cells, in particular neutrophils, in patients with sepsis and septic shock. Neutrophils from septic patients exhibit delayed apoptosis and diminished chemotactic mobility [339,340]. These cells can be activated far from infection sites [341,342]. In septic shock patients, the percentage of immature neutrophils increases. This parameter is associated with a higher risk of lethal outcome after septic shock [340]. 

In septic patients, enhanced values were reported for neutrophil products such as elastase [343], myeloperoxidase [344,345,346], and neutrophil extracellular traps [347,348,349]. Moreover, neutrophil-derived cytotoxic agents, enhanced formation of free heme [350,351,352], dysregulation in the sequestration of transition metal ions [353,354], and dysfunctional mitochondria [355,356,357] were also reported to contribute to damaging reactions in sepsis. 

In sepsis, it is very challenging to predict which protective mechanism will be exhausted first. Several factors contribute to this uncertainty, such as the local protective status in the affected organs, the energy and immune status of patients, existing comorbidities, and genetic predisposition [358]. A personalized analysis of the status of protective systems is mandatory for individual therapeutic approaches. 

## 5. Conclusions

A functioning immune system is mandatory for long-term surveillance of our organism by removing any harm that can disturb the integrity of cells and tissues. Otherwise, the activation of immune cells is associated with the release of aggressive, cell-damaging agents. These host-derived cytotoxic agents are an essential part of the immune response. The interplay between these agents and ready-to-use antagonizing principles determines the further fate of the inflammatory process.

Multiple mechanisms contribute to cell and tissue damage and thus to the development of chronic inflammatory disease states. The permanent damage of unperturbed cells by cytotoxic agents causes again and again the release of alarmins and antigens and the recruitment of novel immune cells. An important pathophysiological aspect in this vicious circle of initiation of immune responses and destruction of biological material is the decline, exhaustion, or inactivation of ready-to-use acting protective mechanisms in the affected tissues. Further complications result from dysregulated synthesis of ECM components, the existence of long-lasting immunocompromised conditions, and the colonization of commensal and mutualistic pathogens in inflamed tissue areas.

Although protective principles can act very efficiently against the corresponding damaging agent, their capacity is limited. As long as damaging agents are only weakly expressed, efficient protection is given, additional protective systems can be induced, and supplementation of consumed systems is working. Problems arise under conditions of severe and long-lasting action of damaging agents. Then, protection is diminished step by step and biological material is progressively damaged.

Both enhancement of oxidative modifications in biological material and increased proteolytic cleavage of substrates are central events in cell and tissue damage initiated by host-derived cytotoxic agents. In the pathogenesis of chronic inflammatory diseases, several types of host-derived cytotoxic agents often act in concert and promote each other. Enhanced formation of reactive species not only affects the release of transition metal ions and heme components, which have a high potential to further promote oxidative reactions, but also contributes to increased proteolytic damage of ECM and other constituents by inactivation of antiproteases, activation of pro-inflammatory peptides, and initiation of disturbances in the synthesis of novel matrix components. Although some links have been shown in activation and inactivation pathways between different cytotoxic agents and their antagonizing principles, we are far from a thorough understanding of the underlying molecular processes. 

It also remains very challenging to determine which cytotoxic agents predominate in damaging reactions in a certain pathology. Moreover, individual expression of protective mechanisms against host-derived cytotoxic agents varies considerably from patient to patient. These uncertainties substantially impede the implementation of personalized therapies based on substitution of inefficient counter-regulating principles for patients with chronic inflammatory diseases. Their successful implementation requires not only the development of novel therapeutic approaches, but also progress in the diagnosis of the individual status of protective mechanisms.

## Figures and Tables

**Table 1:** Major host-derived cytotoxic agents and their antagonizing principles.

Cytotoxic Agent	Mode of Cytotoxic Action	Antagonizing Principles	Remarks
Superoxide anion radical	Release of Fe^2+^ from [4Fe-4S]^2+^ clusters, formation of peroxynitrite	Superoxide dismutases, cytochrome c	
Hydrogen peroxide	Formation of hydroxyl radicals in reaction with Fe^2+^ or Cu^+^	Catalase, peroxiredoxins, glutathione peroxidases	
Hydroxyl radicals	Diffusion-controlled oxidation of many substrates	No antagonizing principles; only limited protection by carbohydrates	Prevention of their formation is the main strategy Very dangerous
Peroxynitrite	Formation of substrate radicals, nitration of tyrosine residues, initiation of lipid peroxidation	Myeloperoxidase, heme proteins	
Hypochlorous acid, hypobromous acid	Preferred oxidation of cysteine, methionine residues Interaction with aromatic amino acid residues and amino groups	SCN^−^, taurine, glutathione (GSH), ascorbate	
Myeloperoxidase (MPO)	Formation of HOCl, HOBr, substrate radicals	Ceruloplasmin	
Free transition metal ions	Dangerous radical species in reaction with H_2_O_2_ and organic hydroperoxides	Proper control over all aspects of iron and copper ion metabolism	Enhanced yield of free transition metal ions is dangerous
Free methemoglobin	Formation of free heme	Haptoglobin	
Free metmyoglobin	Formation of free heme	Haptoglobin	
Free heme	Oxidation at hydrophobic loci, hemolysis of red blood cells, cytotoxic to kidney and liver, interaction with G4 structures in nucleic acids, can act as DAMP	Hemopexin Heme oxygenase	Very dangerous
Oxidative products in lipid phases such as lipid peroxyl radicals and lipid hydroperoxides	Induction of further oxidative modifications of yet-unperturbed molecules	Lipid antioxidants such as α-tocopherol, carotinoids, ubiquinol, dehydrolipoic acid Glutathione peroxidase 4 (GPX4), and GSHProper control over transition free metal ions	
Oxidative products in water-exposed molecules	Induction of further oxidative modifications of yet-unperturbed molecules	Urate, ascorbate, polyphenols Proper control over transition free metal ions	
Neutrophil elastase	Cleavage of many extracellular matrix components, formation of angiotensin II	α_1_-antitrypsin (A1AT), secretory leukocyte protease inhibitor (SLPI), elafin, serpin B1, α_2_-macroglobulin	Failure of anti-proteases to inhibit elastase at severe oxidative stress Very dangerous
Cathepsin G	Cleavage of extracellular matrix components, receptor shedding, formation of angiotensin II	A1AT, α_1_-antichymotrypsin, SLPI	
Proteinase 3	Cleavage of extracellular matrix components, in particular elastin	A1AT, elafin	
Mast cell tryptases	Cleavage of extracellular matrix components	Heparin-binding proteins such as lactoferrin, MPO, antithrombin III	Protected by heparin against the action of anti-proteases
Mast cell chymase	Cleavage of extracellular matrix components, chemokines, and cytokines, formation of angiotensin II	α_1_-antichymotrypsin	
Angiotensin II	Receptor-mediated pro-inflammatory effects	Angiotensin converting enzyme 2 (ACE2)	Very dangerous
Bradykinin	Receptor-mediated pro-inflammatory effects	Aminopeptidase P, angiotensin converting enzyme (ACE)	
Matrix metalloproteases (MMPs)	Cleavage of extracellular matrix components	Tissue inhibitors of metalloproteases (TIMPs)	Problems at shifted balance between MMPs and TIMPs

**Table 2:** Environmental cytotoxic agents and their antagonizing principles.

Cytotoxic Agent	Mode of Cytotoxic Action	Antagonizing Principles	Remarks
Singlet oxygen (^1^O_2_)	DNA damage, especially guanine [21,22]	Carotenoids [23,24,25]	Skin and eye exposure
Ozone	Formation of ozonides and cytotoxic aldehydes [26]	Ascorbate, GSH, urate [27,28]	Exposure to respiratory system [29,30]
Sunlight	Induction of photooxidative processes, formation of ^1^O_2_ [31]	Melanin, polyphenols, [32]	Skin exposure
Ionizing irradiation	Water radiolysis, formation of solvated electrons, O_2_^•−^, H_2_O_2_, ^•^OH, and substrate radicals [33,34,35]	See remarks in Table 1	Always present at very low level

## Data Availability

Data sharing is not applicable to this article.
